# Digital Evidence: Revisiting Assumptions at the Intersection of Technology and Assessment

**DOI:** 10.5334/pme.1270

**Published:** 2024-11-20

**Authors:** Andrew E. Krumm, Saad Chahine, Abigail M. Schuh, Daniel J. Schumacher, Sondra Zabar, Brian C. George, Kayla Marcotte, Stefanie S. Sebok-syer, Michael A. Barone, Alina Smirnova

**Affiliations:** 1 Learning Health Sciences, Surgery, and Information, Medical School and School of Information, University of Michigan, Ann Arbor, Michigan, United States; 2 Faculty of Education, Queen’s University, Canada; 3 Division of Emergency Medicine, Medical College of Wisconsin, Milwaukee, Wisconsin, United States; 4 Division of Emergency Medicine, Cincinnati Children’s Hospital Medical Center/University of Cincinnati College of Medicine, Cincinnati, Ohio, United States; 5 Division of General Internal Medicine and Clinical Innovation at the NYU Grossman School of Medicine, New York, New York, United States; 6 Surgery and Learning Health Sciences, University of Michigan, Ann Arbor, Michigan, United States; 7 Department of Learning Health Sciences, University of Michigan, Ann Arbor, Michigan, United States; 8 Emergency Medicine, Stanford University, Stanford, California, United States; 9 The American Board of Pediatrics, Chapel Hill, North Carolina, United States; 10 Department of Family Medicine, University of Calgary, Calgary, Alberta, Canada

## Abstract

The increasing use of technology in health care and health professions education is an invitation to examine how digital sources of evidence are used in making assessment claims. In this paper, we describe how four sets of terms—primary and secondary data; structured and unstructured data; development and use; and deterministic and generative—can aid in examining how data from digital sources are used in evaluating what learners know and can do. Drawing on multiple examples, this paper shows how the four sets of terms can help both developers and users of technology-based assessment systems.

## Introduction

A new era in medical education assessment is emerging, spurred on by the continued integration of technology into health care. For example, smartphone-based workplace assessments have made it easier than ever for supervising physicians to rate and provide feedback to trainees [1,2]. Instruments and tools used in providing care, such as video recordings of laparoscopic surgical procedures, have provided new opportunities to collect rich data on learners’ developing abilities [3]. In addition, electronic health records (EHRs) have opened up a wealth of data on learners’ experiences across clinical encounters [4,5]. Despite many new technological integrations, the ways in which they have improved assessment practices in medical education remain unique from program to program and technology to technology.

While formal, standardized testing still predominates at key transitions in medical training, more programmatic approaches are proliferating [6,7]. In these newer approaches, information from several sources are combined and triangulated in the service of an educational decision such as remediating or accelerating a learner [8,9]. With novelty comes a need for clear connections between sources of data and intended assessment claims. One reason for this is that novel data do not have the taken-for-granted psychometric properties of data generated from standardized multiple-choice exams. To help connect new sources of data to coherent assessment claims, this paper highlights a set of key terms that have emerged as researchers have worked with new and novel data sources for the purpose of assessment: primary and secondary data; structured and unstructured data; development and use; and deterministic and generative. These terms bring together lessons learned from previous assessment eras while also highlighting potential affordances and constraints of using new sources of data to support educational decision-making. More broadly, these terms are intended to provide a framework for future conversations, helping medical educators anticipate and capitalize on evolving opportunities in the form of a shared vocabulary [10].

Modern conceptions of assessment involve reasoning from one or more specific pieces of evidence to a claim about what an individual knows or can do more generally [11,12]. The ways in which specific pieces of evidence are collected, summarized, and reported on are central to the strength of the statements and overall argument one can make about an individual’s general knowledge, skills, or abilities. With the changing landscape of assessment, revisiting the meaning behind words used in developing assessment arguments is both timely and appropriate. Below, we describe four pairs of terms. Each pair is intended to improve the precision with which new and novel pieces of evidence are connected to assessment claims in health professions education.

### Primary and Secondary Data

Previous (and continuing) assessment paradigms have based their epistemological foundations on controlled, prospective data collection in line with the following steps: assessment designers begin with a construct of interest (e.g., medical knowledge) and design assessment tasks (e.g., standardized tests) that are explicitly designed to elicit the construct [13]. The outcomes of these designed tasks can then be used to characterize the degree to which a learner possesses some knowledge, skill, or ability. In the best of cases, the assessment tasks in which learners are engaged are well suited to generate evidence for a target of measurement (i.e., constructs) and that resulting evidence can be directly linked to an educational decision [14]. As new technologies deposit ever increasing amounts of data, however, the prospective flow from targeted constructs to evidence used for assessment purposes is being reversed. Framed in question form: there is so much data available in the electronic health record (EHR), how can medical educators repurpose these data to measure a learner’s knowledge or abilities?

Based on questions prompted by new technologies, we propose clearly delineating between **primary** and **secondary** data, especially when considering an assessment system and its potential use [15]. Primary data can be thought of as being collected for a specific assessment purpose from the outset, where an assessment developer defines a construct of interest and identifies appropriate tasks that could elicit the construct (i.e., prospective data collection). By contrast, secondary data means data that has been previously generated for different reasons and then later used to support an assessment claim. The work of Schumacher et al. demonstrates how data generated from an EHR, for example, was used to generate “Resident Sensitive Quality Measures” [16]. Using data for secondary assessment purposes can require significant work around isolating useful data elements. Schumacher et al., for example, first used a Delphi approach to determine metrics that could be gleaned from the EHR followed by a quantitative analysis of the data to determine whether these metrics could be used to differentiate residents based on their clinical performance [17,18]. Because secondary data are, by definition, collected for another purpose, a broad array of approaches need to be used in order to build coherent evidentiary arguments. We highlight five approaches that developers can use in transforming secondary data for assessment purposes:

1. Facilitating a collaborative, Delphi approach where knowledge from experts and front-line individuals is combined to align available data, analytical approaches, and reporting metrics to a desired construct;
2. Observing a behavior outside of a digital system and developing a predictive model based on digital data to later predict the behavior;
3. Using logic as well as frontline and expert opinion to identify steps that a user *should* follow in a digital system from which data are collected—following identified steps, therefore, can be quantified as “good” and deviations as “bad;”
4. Reverse engineering a construct using a structured assessment process like Evidence-Centered Design, whereby secondary data are aligned to an intended construct, additional irrelevant factors are specified, the influence of clinical tasks are described, and strategies for translating available data into interpretable and usable scores are documented; and
5. Rating/coding raw, existing data in terms of the presence or absence of an intended construct [19].

As a form of evidentiary reasoning, working with secondary data requires clear evidence rules, i.e., what should a group of medical educators be allowed to use in their deliberations on a learner [11,13]. The quality of the underlying data collection approach, the soundness of the data analysis, and the interpretability of a resulting metric all must be considered when using a resulting score as part of a broad assortment of evidence used in rendering a competency determination. For many potential sources of data, like a standardized test, collection, analysis, and reporting are presumed and readily used in supporting a broader determination of competency. What count as scores that can serve as evidence from data generated from an EHR or clinical notes have not yet reached the same degree of wide acceptance. A wide level of acceptance is importantly tied to the stakes associated with the educational decision. For example, Schaye et al. demonstrated an approach for assessing residents’ clinical notes and Sebok-Syer et al. showed that clinical performance metrics are more readily accepted by residents and faculty as being “objective,” but in this instance the focus was formative and not summative [20,21]. Key to increasing the use of secondary data, therefore, will be increasing numbers of examples and success stories as well the frequency by which these data accompany assessment claims.

### Structured and Unstructured Data

**Structured data** are the result of a pre-defined, tabular format (i.e., rows, columns, and cells). Structured data can be clean or messy in tabular form; examples include medications and tests ordered by a provider extracted from the EHR [22]. These data can be readily analyzed using statistical software. **Unstructured data**, on the other hand, is not initially structured in a pre-defined, tabular format (e.g., video, audio, and images). Unstructured data can also include text-based data like essays, workplace-based assessments (WBA) narrative comments, or patient notes from an EHR [23,24]. Both primary and secondary data can be either structured or unstructured. Structure can come from the instruments used to collect assessment data like rubrics or tests. Likewise, rater-mediated assessments often involve structured observation protocols [25]. For more unstructured data (i.e., not initially tabular) like video, audio, or free-form text, structure needs to be applied in order to analyze what is collected. When and how that structure is applied is a critical element of any assessment argument because the application of some rules that place complex human behavior into tabular form necessarily simplifies that behavior, and this simplification can have a variety of consequences as an assessment argument is used to support educational decisions or actions [23]. Assessment involves “bracketing” the world into structured forms, and the nature of that bracketing can be more qualitative when developing different categories, transition to more quantitative through rubrics and scales, and resolve in a categorical distinction like “ready or not ready for independent practice” [26]. Questioning the timing of structured/unstructured data as well as qualitative/quantitative data and the meaning and boundaries around what is meant by assessment is a critical activity for nearly all assessment developers [27].

Table 1 lays out different combinations of primary, secondary, structured, and unstructured data. Key to working with both structured and unstructured data is identifying when structure is applied (e.g., in an instrument’s design or in simplifying continuous data streams) and the potential effects this can have on how these assessment data are used.

**Table 1:** Relationship between Primary and Secondary Data and Structured and Unstructured Data.


		SOURCE	

		PRIMARY	SECONDARY

Organization	**Structured**	Tests, WBA ratings, Objective Structured Clinical Examination checklists	Discrete EHR data elements, patient satisfaction surveys, discrete variables in templated clinical notes

	**Unstructured**	Video/audio of clinical interaction, WBA narrative comments, Discharge notes	General clinical notes from EHR


**Note:** WBA = workplace-based assessments.

### Development and Use

When used to support specific educational decisions, individual assessments can be thought of as one part of a longitudinal process. Often, however, phrases like, “this instrument is validated” predominate and stand in for complex interactions among people, tools, and organizational processes that make collecting and acting on data possible. Moreover, many concepts in assessment, such as reliability, tend to be used in the context of one phase of an assessment’s life—its **development**. Questions like feasibility of completing an instrument, the technological tools needed for a scoring approach (e.g., computer adaptive tests), and settings in which users will actually interact with a score report at a particular time (e.g., competency committee meeting) center on a second phase of an assessment system—its **use**. A key lesson from clinical research that leverages new sources of data is recognizing that developing a quantitative tool is only part of the process. In fact, developing the tool is the easy part; reliably implementing the tool is far harder [28,29].

Across primary, secondary, structured, and unstructured data, the process of translating these data for particular users (e.g., learner, faculty, program leader) to support a specific decision (e.g., competency determination or recommendations on what to work on next) *is* the work of assessment. Developing an assessment involves identifying when and under what conditions data are collected, how various elements from an assessment will be combined, and how a resulting score will be reported and shared with an end user [30]. *Development* is when issues of validity (e.g., coherent argument and/or correlation to desired outcomes) and reliability (e.g., reproducibility and interrater reliability) are central [31]. St. Onge et al. argue for the importance of connecting analyses carried out during the development of an assessment system (e.g., correlation and reliability coefficients) with the purpose of an assessment [32]. Focusing on purpose helps to refocus assessment developers on potential users, the day-to-day decisions they need to make, and how the previously developed data collection, analysis, and reporting processes help users accomplish their goals. Therefore, use brings about broader conceptions of validity and calls attention to concrete operational concerns. Said differently, use directly implicates implementation: Will this assessment system work here? Do we have the right infrastructure? Do individuals have the right training to deploy the assessment and use the score appropriately? Just because an assessment system has accrued positive evidence during its development does not mean that it will be readily integrated into regular practice or that when implemented it will replicate previous results.

### Deterministic and Generative

A typical assessment process spans the activities of collecting, analyzing, and reporting on data for the purpose of supporting claims about learners. Recent advances in generative artificial intelligence have brought forward new opportunities for analyzing assessment data and thus new ways of thinking about a typical assessment process. Importantly, the outputs of the analysis step can differ dramatically when either deterministic or generative models are used. Nearly all analytical models used for assessment can be called deterministic. While this term has many different meanings within different analytical frameworks, when paired with the term generative, deterministic refers to the property of an analytical model where the same inputs will lead to the same outputs. Generative models, such as large language models, on the other hand, will produce different outputs even when the same inputs are used (the term “stochastic” from the phrase “stochastic parrots” captures the non-deterministic output of these models and is often used to counterbalance hype around large language models as artificial intelligence) [33].

The ways in which deterministic versus generative models are developed can play an important role in their eventual use for assessment purposes. For example, most psychometric models, whether complex statistical models or simple weighted average calculations, are calibrated on a representative sample of learners. Calibration is not just about learning parameters and algorithms that will be used for subsequent scoring; it helps in clarifying the assessment tasks themselves in terms of adapting, adopting, or abandoning different tasks. Generative models, by contrast, are learned at such a large scale that users of these models for assessment purposes cannot directly modify scoring parameters or drop entire tasks or items. Instead, users of these types of models develop highly specific prompts or implement bespoke model refinements that help the generative model produce more desirable, consistent responses.

Deterministic versus generative models also differ in how the analytical model, when applied to a set of inputs, produces an output. Each step in this process is knowable from a deterministic perspective. Even the most complex computer adaptive testing systems can be understood at each step of item presentation, scoring, and subsequent selection. This knowability is currently not possible for generative models. When assessment scores are attached to consequential stakes (e.g., certification), knowability is a critical asset. Generative models, therefore, may be best suited to contexts where the outputs of the model need not be a deterministic score but a distillation or summarization of complex unstructured data with low to no stakes attached.

Across primary and secondary data, structured and unstructured data, development and use, as well as deterministic and generative models we have organized Table 2 to illustrate relationships between key terms and example assessments.

**Table 2:** Relationships between Example Assessments and Key Terms.


	EXAMPLE ASSESSMENTS		

KEY TERMS	TRADITIONAL TEST *(LICENSURE, CERTIFICATION)*	SHORT, PRACTICAL TOOL *(WBA)*	CLINICAL *(EHR)*

Primary and secondary	Intentional data collection (prospective) from standardized settings Characterized by infrequent administration, multiple items per instrument	Intentional data collection (prospective) from opportunistic settings Characterized by frequent administration, few items per instrument	Opportunistic collection (repurposed) of preexisting data from complex settings Characterized by emphasis on machine-executable operationalizations from clinical record

Structured and unstructured	Highly structured	Both highly structured (e.g., ratings) and unstructured (e.g., written comments)	Both structured (whether a medication was ordered) and unstructured (e.g., clinical notes)

Development and use	Well understood instrument development processes, known summarization strategies (e.g., CTT, IRT), taken-for-granted score reporting, often clear summative and formative uses, relatively easy to implement	Developing summarization strategies, developing score reporting approaches, used for both formative and summative purposes, more challenging to implement	Developing summarization strategies, available touchstones for reporting (e.g., QI audit and feedback), typically used to document that an action occurred, development is high burden, but use may be easier because operations happen in the “background”

Deterministic and generative	Well understood deterministic models	Both deterministic and generative models whereby generative models can be used to summarize multiple feedback encounters into a holistic statement	Deterministic for summarizing more structured data and generative models for summarizing more unstructured data elements


**Note:** CTT = classical test theory; IRT = item response theory; QI = quality improvement.

## Concluding Thoughts

The purpose of this Eye Opener is to outline terms where shared understanding may be needed in the next era of assessment. Given the ways in which assessment is about reasoning from specific evidence to more general claims about what a learner knows and can do, clarity around primary and secondary data, structured and unstructured data, development and use, and deterministic and generative models will help in strengthening future assessment arguments. While existing frameworks like Evidence-Centered Design will and should continue to play a primary role in developing coherent assessment arguments, the terms introduced in this paper are intended to enhance communication among those developing one or more arguments [14]. In addition, the terms highlighted in this paper point to opportunities for innovation and future research by specifying tradeoffs with different data types and analytical models as well as the different activities involved in developing and using assessments.

While this Eye Opener seeks to advance descriptions of “how” for the next era of assessment, assessment is also grappling with “why”: Why collect these data? Why use these data in these ways? As the next era unfolds, more and more attention may be directed toward patients and their experiences and not just the performance of the developing professional. A critical challenge in using any assessment system is that current approaches to assessment, across the continuum, are not sufficiently aligned with patient outcomes. As new assessments are developed to cover evermore skills, evidentiary arguments will continually need to be evaluated considering myriad downstream outcomes.
